# Characteristics and Epidemiological Investigation of Paratuberculosis in Dairy Cattle in Tai'an, China

**DOI:** 10.1155/2020/3896754

**Published:** 2020-03-13

**Authors:** Zilong Cheng, Mengda Liu, Peng Wang, Peng Liu, Meng Chen, Jiandong Zhang, Sidang Liu, Fangkun Wang

**Affiliations:** ^1^College of Veterinary Medicine, Shandong Agricultural University, Tai'an, Shandong, China; ^2^Shandong Provincial Key Laboratory of Animal Biotechnology and Disease Control and Prevention, Shandong Agricultural University, Tai'an, Shandong, China; ^3^Laboratory of Zoonoses, China Animal Health and Epidemiology Center, Qingdao, Shandong, China

## Abstract

Paratuberculosis, a chronic and sometimes fatal disease of ruminants, is caused by *Mycobacterium avium* subsp. *paratuberculosis* (MAP). In this study, we examined paratuberculosis cases among 2–4-year-old dairy cows at farms in Shandong Province, China. Paratuberculosis cases were diagnosed based on clinical symptoms, pathological autopsy, and histopathological inspection. Characteristics of paratuberculosis in the affected dairy cattle included poor body condition, persistent diarrhea, subcutaneous edema, granulomatous ileitis (multibacillary), mesenteric lymphadenitis, and hepatitis. Acid-fast bacilli from fecal specimens and lymphocytes were putatively identified as MAP based on Ziehl-Neelsen staining, then confirmed using polymerase chain reaction-based testing and matrix-assisted laser desorption/ionization time-of-flight mass spectrometry analyses. Overall, only one MAP strain was isolated from a herd with symptomatic diarrhea. However, analysis of 586 serum samples from nine herds in Tai'an City revealed that 66.7% of herds and 14.2% of animals were seropositive for MAP. Our findings suggest that paratuberculosis is widely prevalent and therefore a significant threat to the dairy industry in Tai'an City, Shandong Province, China.

## 1. Introduction

Paratuberculosis, also known as Johne's disease, is a contagious, chronic, and sometimes fatal infection that primarily affects the small intestine of ruminants [1–4]. It is caused by *Mycobacterium avium* subsp. *paratuberculosis* (MAP). Clinical symptoms of paratuberculosis include slowly progressive wasting and diarrhea, which are intermittent at first but become progressively more severe until they are constantly present. Affected animals become increasingly emaciated and usually die as a result of dehydration and severe cachexia. Paratuberculosis has also been reported in horses, pigs, rabbits, stoats, foxes, and weasels [5].

MAP has been isolated from milk and colostrum from naturally infected dairy cows [6]. However, the isolation of MAP from pasteurized whole milk and bovine muscle tissues is a major concern as it shows the disease could potentially be transferred to other cows and even humans [7, 8]. Interestingly, paratuberculosis shows similarities to Crohn's disease in humans [9], suggesting that MAP is potentially a causative agent of Crohn's disease [10–14]. MAP-positive herds experience an economic loss of almost US$100 per cow compared with MAP-negative herds as a result of reduced milk production and increased animal replacement costs. As such, paratuberculosis is responsible for considerable economic losses within the dairy industry in the United States, estimated at more than $200 million per year [15]. Therefore, bovine paratuberculosis draws significant attention.

Shandong Province in China has a thriving cattle industry, with many of the largest dairy farms in China [16, 17]. However, there are few reports on the epidemiology of paratuberculosis in this region, as well as an overall lack of information regarding disease characteristics and histopathologic changes resulting from MAP infection. During September and October 2016, persistent diarrhea that was resistant to antibiotic treatment was reported in several cows at two dairy farms in Tai'an City, Shandong Province. As a result, the cows became very emaciated. Based on clinical inspection, histopathological examination, and etiological methods, we verified that these two cases were positive for paratuberculosis. In addition, MAP was successfully isolated from the affected cattle. We then carried out more widespread serotesting for MAP infection across nine different dairy herds to gain a better understanding of the prevalence of paratuberculosis in the region and to assess whether this communicable disease represents a significant threat to the dairy industry in Shandong Province.

## 2. Materials and Methods

### 2.1. Ethics Statement

All protocols and procedures were performed according to the Chinese Regulations for Laboratory Animals (State Council of the People's Republic of China, available at http://www.gov.cn/gongbao/content/2017/content_5219148.htm). The study was approved by the Committee on Animal Ethics of Shandong Agricultural University. Experiments were carried out in accordance with the approved guidelines (No. SDAUA-2016-004).

### 2.2. Cases and Sample Collection

During September and October 2016, five dairy cows aged 2 (*n* = 2) or 3 (*n* = 3) years were prematurely culled at two separate farms in Tai'an City, Shandong Province, China. One herd consisted of 550 cows and the other had 536 cows. The diagnosis of the case and the isolation and identification of the pathogen were based on the methods reported in a previous study [18]. The five cows were euthanized because they were extremely thin. They showed a positive MAP antibody test, and their stool samples were polymerase chain reaction- (PCR-) positive for MAP. Stool samples were collected from two farms, where several other cows within the herds were also suffering from persistent diarrhea. The samples were stored at 4°C for up to 48 h prior to processing for culture and DNA extraction.

### 2.3. Preparation of Tissue Samples for Microscopic and Molecular Examination

The test was performed as per the method of Yue et al. [18]. Following euthanasia, the gastrointestinal tracts and mesenteric lymph nodes were collected from two of the five cows for postmortem examination. Various segments of the intestines, mesenteric lymph nodes, and other tissues were collected and separated into two parts. One was fixed in 10% formalin solution for histopathological examination, while the other was stored at −80°C for DNA extraction.

### 2.4. Staining of Fresh Stool Samples and Tissue Samples

Fresh stool samples and mesenteric lymph nodes were smeared on sterile glass slides and stained using the Ziehl-Neelsen method [19]. Stained samples were then examined by light microscopy for the presence of acid-fast bacilli. Formalin-fixed and paraffin-embedded tissues were routinely sliced into 4 *μ*m thick sections. These tissue sections were then stained with hematoxylin and eosin for microscopic examination or using the Ziehl-Neelsen method for detection of acid-fast bacilli.

### 2.5. PCR-Based Detection of MAP

A PCR-based method was used to detect the presence of MAP in various samples. Primers P90 forward (5′-GAAGGGTGTTCGGGGCCGTCGCTTAGG-3′) and P91 reverse (5′-GGCGTTGAGGTCGATCGCCCACGTGAC-3′) were used in amplifying a 413 bp fragment of multicopy insertion element IS*900* as previously described [20]. Thermal cycling was performed using an initial denaturation step of 95°C for 5 min, followed by 30 cycles at 95°C for 30 s, 62°C for 30 s, and 72°C for 1 min, with a final elongation step at 72°C for 10 min.

### 2.6. Isolation and Identification of MAP

A modified centrifugation method was used to cultivate MAP as described previously [21, 22]. Briefly, 3 g aliquots of fecal samples that were tested positive for MAP by PCR and Ziehl-Neelsen staining were added to 30 ml of sterile distilled water. The tubes were vortexed for 2 min and then allowed to stand undisturbed for 30 min. Aliquots (5 ml) of the supernatant were then added to 35 ml of 0.9% (*w*/*v*) hexadecylpyridinium chloride (Sigma, USA) and allowed to stand undisturbed for 12 h at room temperature for decontamination. Tubes were centrifuged at 1500 × *g* for 30 min, and the supernatants were decanted. The remaining cell pellets were resuspended in 1 ml of antibiotic mixture (50 *μ*g/ml amphotericin B, 100 *μ*g/ml nalidixic acid, and 100 *μ*g/ml vancomycin). Cell suspensions were then incubated for 12 h at 37°C before 0.2 ml volumes were inoculated on Herrold's egg yolk agar supplemented with mycobactin J and ANV (Becton Dickinson, USA). They were incubated in sealed 25 cm^3^ tissue culture flasks at 37°C for 6 weeks. Colonies that formed within 1 week were abandoned. Colonies with typical MAP morphology were checked by Ziehl-Neelsen staining and IS*900* PCR as described above. For staining, single colonies were resuspended in 200 *μ*l of sterile water and smeared onto clean glass slides.

Total DNA was then extracted from putative MAP colonies using a TIANamp Bacteria DNA Kit (TIANGEN Biotech, China) according to the manufacturer's instructions and used as a template for PCR-based MAP verification as described above.

Ziehl-Neelsen stain- and PCR-positive colonies were then confirmed as MAP using matrix-assisted laser desorption/ionization time-of-flight mass spectrometry (MALDI-TOF MS) [23]. Briefly, colonies were directly transferred to an MSP 384 target polished steel BC plate. A 1 *μ*l volume of 70% formic acid was spotted, air dried, and covered with 1 *μ*l of HCCA matrix (acyano-4-hydroxycinnamic acid in 50% acetonitrile-2.5% trifluoroacetic acid; Bruker Daltonics, Germany). MALDI-TOF MS identification was performed using a Microflex LT Smart MALDI-TOF mass spectrometer, MALDI Biotyper software version 4.0, and the MBT Compass Library (DB-7854). A log score of >2.0 was considered sufficient for identification at the species level.

### 2.7. DNA Characterization of MAP Isolates

The IS*1311* PCR restriction endonuclease analysis (REA) was performed as described previously, to identify the strain type of MAP [20, 24–26]. Briefly, the extracted DNA was added in an IS*1311* PCR mix containing 1 *μ*M of primers M-56 (5′-3′ GCGTGAGGCTCTGTGGTGAA) and M-119 (5′-3′ ATGACGACCGCTTGGGAGAC) and Taq Plus Master Mix II Dye Plus (Vazyme Biotech Co., Ltd., China). 8 *μ*l of the positive IS*1311* PCR solution was digested for 2 h at 37°C in a 16 *μ*l reaction, which contained 2 U of each endonuclease *Hinf*I and *Mse*I supplemented with buffers provided by the manufacturer (Takara, Japan). And the products were analyzed by gel electrophoresis with 2% agarose gel and observed under UV light (Alpha RED, ProteinSimple).

### 2.8. Enzyme-Linked Immunosorbent Assay (ELISA)

To determine the prevalence of MAP in dairy cows and on farms in Tai'an City, 586 serum samples were collected from cows from nine dairy farms, including the two farms mentioned above, in Tai'an City, Shandong Province. The samples were examined using paratuberculosis ELISA test kits (IDEXX, USA) as per the manufacturer's instructions. Sera with *S*/*P* ratios ≤ 0.45 were scored as MAP-negative, while ratios ≥ 0.55 were considered positive. Intermediate *S*/*P* values were scored as “suspect” and treated as negative for the data analysis.

## 3. Results

### 3.1. Clinical Symptoms and Necropsy

**Figure 1:**
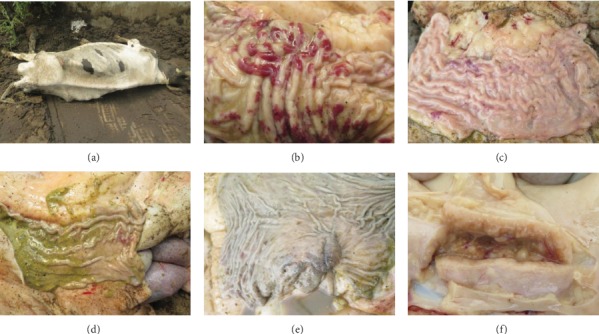
Gross lesions of the infected cows. (a) The affected animal is emaciated. (b, c) Gyriform swelling, mucopurulent discharge, and hemorrhaging of the ileum mucosa. (d) Gyriform swelling and hemorrhaging of the cecum. (e) Focal ulceration on the ileocecal valve. (f) Significant medullary swelling of the mesenteric lymph nodes.

Affected cows initially exhibited persistent diarrhea without other obvious symptoms. All cows had a normal body temperature, and their mental status was considered good. Diarrhea samples appeared homogeneous, nonhemorrhagic, and nonmucoid with no other inflammatory secretions. Diarrhea could not be controlled with antibiotics. From one to several months after the onset of persistent diarrhea, the predominant symptoms displayed by infected cattle included decreased milk production and chronic progressive weight loss with chronic or intermittent diarrhea. In the late stage of the disease, the affected cattle were emaciated, with obvious atrophy of skeletal muscle and fat (Figure 1(a)).

Necropsy of the euthanized cows revealed lesions in the gastrointestinal tract accompanied by swelling and thickening of the intestinal wall, with the most serious lesions occurring in the small intestine. The walls of the ileum and cecum showed thickening, with gyriform swelling and hemorrhaging of the mucosa. The intestinal mucosa was also corrugated and thickened (Figures 1(b)–1(d)). Focal ulcers were observed on the ileocecal valve (Figure 1(e)). Mesenteric lymph nodes were significantly enlarged and elongated. Lymph node sections showed medullary swelling and an outflow of pus-like material (Figure 1(f)). All gross lesions observed during necropsy of the infected animals coincided with the lesions typical of paratuberculosis.

### 3.2. Presence of Acid-Fast Bacilli in Feces and Lymphocytes and following Pure Culture

**Figure 2:**
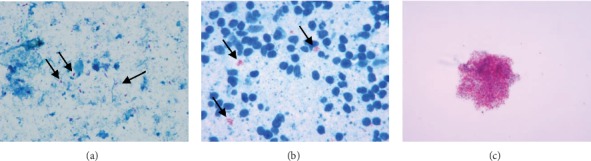
AFB in the feces, lymphocytes, and pure culture stained by the Ziehl-Neelsen method. (a) A small amount of red AFB in the feces (arrows); AFB, 1000x. (b) Some AFB in the smears of lymphocytes (arrows); AFB, 1000x. (c) A large pure AFB clump stained by Ziehl-Neelsen; AFB, 1000x.

Multiple acid-fast bacilli were observed in smears prepared from fecal samples and lymphocytes, as well as in pure cultures. While some scattered acid-fast bacilli were observed in the fecal samples (Figure 2(a)), most bacilli were clustered into masses or inside cells in the lymphocyte samples (Figure 2(b)). A large clump of acid-fast bacilli was observed in the pure culture (Figure 2(c)).

### 3.3. Microscopic Examination

**Figure 3:**
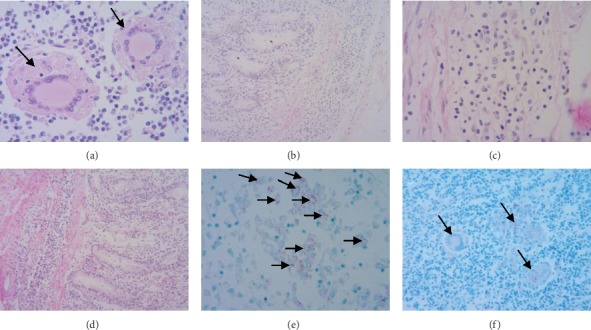
Pathological changes of the diseased cows. (a) Multinuclear giant cells (arrows) in the mesenteric lymph nodes; HE, 400x. (b) Infiltration of lymphocytes and macrophages into the lamina propria of the jejunum; HE, 100x. (c) Infiltration of hyperplastic lymphocytes, macrophages, and epithelioid cells into the ileal lamina propria and submucosa; HE, 400x. (d) Infiltration of hyperplastic lymphocytes, macrophages, and epithelioid cells into the cecum lamina propria and submucosa; HE, 100x and 400x. (e) There is an abundance of AFB (arrows) in the epithelioid cells of the ileal lamina propria; AFB, 400x. (f) There are many AFB (arrows) in the multinuclear giant cells of the mesenteric lymph nodes; AFB, 400x.

Lymphopenia was observed in the mesenteric lymph nodes, although large numbers of epithelioid cells and multinuclear giant cells were observed (Figure 3(a)). Hyperemia was noted in the lamina propria of the jejunal mucosa, along with infiltration of lymphocytes and macrophages (Figure 3(b)). Multiple lymphocytes, macrophages, and epithelioid cells were observed in the submucosa of the ileum and cecum (Figures 3(c) and 3(d)). Ziehl-Neelsen staining showed that acid-fast bacilli were present within macrophages and epithelioid cells in the lamina propria of the ileum (Figure 3(e)). Similarly, multiple acid-fast bacilli had infiltrated multinuclear giant cells in the mesenteric lymph nodes (Figure 3(f)).

### 3.4. PCR- and MALDI-TOF MS-Based Identification of MAP

PCR analysis revealed that 5/28 fecal samples were positive for MAP. The amplified fragments were 413 bp in length. The suspected MAP colonies cultured on selective media were also subjected to PCR-based identification. The resulting positive colonies were then confirmed using MALDI-TOF MS. The resulting scores were all >2.0, confirming that the colonies were MAP.

### 3.5. Identification and Strain Typing of MAP

For identification of the strain type, IS*1311* PCR was conducted and followed by digestion by two restriction endonucleases (*Hinf*I/*Mse*I). IS*1311* PCR-REA reaction products confirmed the presence of bands of 67, 218, 285, and 323 bp (Supplementary Fig. [Supplementary-material supplementary-material-1]), which indicated that the isolates were type C.

### 3.6. Prevalence of Paratuberculosis in Tai'an City

A total of 586 serum samples collected from nine dairy farms were tested by ELISA. Overall, 6/9 farms were positive for MAP. For these six farms, 25% (14/56), 6.9% (4/58), 24.2% (32/132), 18.5% (5/27), 9.6% (9/94), and 42.2% (19/45) of samples tested positive for MAP, corresponding to a prevalence of 14.2% across all tested cows. All prevalence results are shown in Table 1.

## 4. Discussion

There were very few reports on the characteristics and epidemiology of paratuberculosis in dairy cows in China, as well as a general lack of detailed descriptions of typical pathological changes resulting from MAP infection. Here, we provided a detailed description of the pathological findings from typical, naturally occurring paratuberculosis cases. The intestinal mucosa of infected animals, including the jejunum, ileum, cecum, and colon, was corrugated and thickened. Mesenteric lymph nodes showed medullary swelling, and consistent with previous reports, a large number of multinuclear giant cells, epithelioid cells, and macrophages were present in the diseased tissues. However, unlike other studies [27], we noted lesions throughout the ileocecum, jejunum, and colon. The lesion was much more severe in these cases compared with previous reports.

Paratuberculosis is always difficult to control because of its chronic feature and the large percentage of subclinical infections [28]. Furthermore, it has been hypothesized that progression in clinical symptoms is a result of a shift from potentially protective cell-mediated immune responses to a nonprotective antibody response [29]. The antibodies against MAP could be found after a long-term infection. During the incubation period, asymptomatic animals can shed MAP to the environment, resulting in further transmission of infection in the herd. Therefore, for each clinically diseased animal, there might be 15 to 20 animals infected within the herd [28]. In the study, the possible primary reason for the local paratuberculosis outbreak was that many cows had been latent infections. So we should pay more attention to the asymptomatic and subclinical animals that can shed MAP. The stress of calving decreased immunity, allowing the proliferation of MAP in infected cows and the development of clinical symptoms such as diarrhea, decreased milk production, and weight loss. Weight loss in paratuberculosis-infected ruminants is a consequence of protein malabsorption and loss caused by the cellular infiltration and edema that occur in the intestine [30]. Infected cows also increased susceptibility to other problems such as mastitis, elevated cell counts, and lameness [31]. In the late stage of the disease, cows became extremely thin and milk production declines sharply. In this study, the identified MAP strain was type C (dairy cattle), consistent with Yue et al.'s previous research [18]. It indicates that type C is the predominant type in cattle in Shandong Province, China. It was consistent with most other previous studies, and type C is the predominant type in the world [24, 25, 32–40].

The seroprevalence investigation indicated that 66.7% (6/9) of herds and 14.2% of animals were positive for MAP antibodies, which is higher than the findings from previous studies from Shandong [18] and other provinces in China [41–44]. These studies recorded MAP antibodies in 2.8%–18% of animals [41–44]. However, we noted significant variation in the prevalence of MAP among the different farms, with percentages of seropositive samples ranging from 6.9% to 42.2%. In general, only 25%–30% of infected animals are detected by even the most sensitive molecular testing techniques [45]. Interestingly, the paratuberculosis cases examined in this study appeared to be more severe than has previously been reported in China. Therefore, the situation regarding paratuberculosis may be more serious in Shandong Province than in other areas of China, which warrants further investigation.

Based on our findings from the epidemiology survey, we suspected that dairymen did not pay enough attention to the prevention and control of paratuberculosis in Shandong Province. Most of them did not have a clear idea about this disease. Even if some symptoms occurred, they did not expect it was caused by paratuberculosis. Normally, the potentially infected cows were not in quarantine, even new cattle were introduced into the farms. Lack of taking effective prevention and control measures against latent infection may be another cause of the outbreak. Due to the limited samples, these data cannot build the national picture. However, they would serve as a warning to the Chinese cattle industry because Shandong, as a traditional cattle-rearing area, was still short of measures to prevent paratuberculosis.

## 5. Conclusions

This study provided a description of the pathological changes associated with paratuberculosis in naturally infected dairy cows in China. It indicated that paratuberculosis was an important infectious disease of dairy cattle in Shandong Province. Appropriate herd management practices, including separation and/or culling of infected animals, should be introduced in a timely manner as standard practice in the dairy industry. On-site veterinarians should pay more attention to this disease as well. Effective management practices are important to reduce both economic and stock losses associated with paratuberculosis and to reduce the risk of disease transmission to humans.

## Figures and Tables

**Table 1:** Information on the selected herds.

Herd ID	No. of animals	Sampling no.	The no. of positives	Positive rate
1	920	56	14	25.0%
2	520	58	4	6.9%
3	874	132	32	24.2%
4	536	27	5	18.5%
5	630	94	9	9.6%
6	550	45	19	42.2%
7	420	39	0	0%
8	950	85	0	0%
9	450	50	0	0%
Total number	5850	586	83	14.2%

## Data Availability

The data used to support the findings of this study are available from the corresponding author upon request.

## Supplementary Materials

Supplementary Materials For identification of strain type, IS*1311* PCR was conducted and followed by digestion by two restriction endonucleases (*Hinf*I/*Mse*I). IS*1311* PCR-REA reaction products confirmed the presence of bands of 67, 218, 285, and 323 bp (Supplementary Fig. 1), which indicated that the isolates were type C.
