# Supplementary figures and images for: Characteristics and Epidemiological Investigation of Paratuberculosis in Dairy Cattle in Tai'an, China
Source: Biomed Res Int. 2020 Mar 13;2020:3896754. doi: 10.1155/2020/3896754 (PMC7094201; doi:10.1155/2020/3896754)

Supplementary figure 1

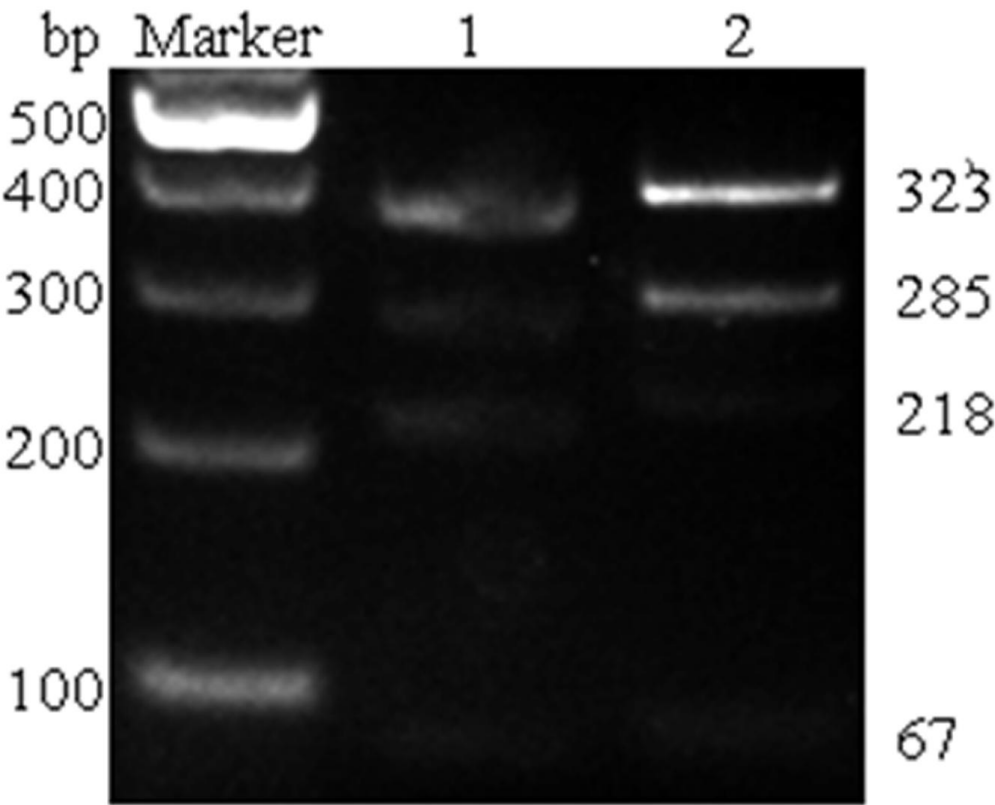

Supplement: Supplementary Materials — For identification of strain type, IS1311 PCR was conducted and followed by digestion by two restriction endonucleases (HinfI/MseI). IS1311 PCR-REA reaction products confirmed the presence of bands of 67, 218, 285, and 323 bp (Supplementary Fig. 1), which indicated that the isolates were type C. [file 3896754.f1.pdf]
